# Artificial Intelligence in Traumatic Brain Injury: A Systematic Review of Prognostic, Diagnostic, and Monitoring Applications

**DOI:** 10.7759/cureus.96600

**Published:** 2025-11-11

**Authors:** Anas E Ahmed, Rayan M Alyami, Fatimah H Al Ghazwi, Renad H Hamzi, Nawa K Alshammari, Fawziah M Jali, Abdullah A Al Alduwayh, Thikra M Almujami, Abdullah S Alamri, Jamal A Sabban, Ghadi F Alsum

**Affiliations:** 1 Community Medicine, Jazan University, Jazan, SAU; 2 General Practice, College of Medicine, University of Bisha, Bisha, SAU; 3 General Practice, College of Medicine, Jazan University, Jazan, SAU; 4 General Practice, College of Medicine, Hail University, Hail, SAU; 5 General Practice, College of Medicine, King Saud University, Riyadh, SAU; 6 General Practice, Faculty of Medicine, Umm Al-Qura University, Makkah, SAU

**Keywords:** artificial intelligence, diagnosis, machine learning, prediction, prognosis, traumatic brain injury

## Abstract

Traumatic brain injury (TBI) remains a leading cause of death and disability worldwide, with outcomes that are highly heterogeneous and difficult to predict using conventional clinical tools. Artificial intelligence (AI) has emerged as a promising approach to enhance diagnosis, prognostication, and management of TBI across diverse care settings.

Following PRISMA guidelines, a comprehensive search of PubMed, Scopus, Web of Science, and Cochrane CENTRAL was conducted from inception to September 2025. Eligible studies applied AI or machine learning techniques to TBI populations for diagnosis, outcome prediction, monitoring, or screening, with data extracted on study design, population characteristics, data sources, model architecture, comparators, and performance metrics. Methodological quality and risk of bias were assessed using the PROBAST tool.

From 10,710 records identified, 12 studies met the inclusion criteria. Prognostic models across emergency triage, intensive care unit, and registry datasets achieved AUCs (area under the curve) between 0.81 and 0.93, with simple logistic regression models often performing comparably to more complex machine learning methods. Imaging-based approaches, including convolutional neural networks for CT segmentation, improved prediction of therapeutic intensity but were constrained by small sample sizes and inconsistent segmentation quality. Unsupervised clustering revealed clinically meaningful phenotypes, though generalizability was variable. Pediatric applications were exploratory, with small cohorts prone to overfitting. Screening models for mild TBI demonstrated overall accuracies of 0.80-0.86 but only modest sensitivity (~0.70) for CT-positive cases. Across domains, interpretability strategies such as SHAP values and fuzzy rules showed promise; however, many studies were limited by inadequate external validation, incomplete handling of missing data, and small or retrospective single-center designs.

Overall, AI applications in TBI demonstrate strong potential for improving prognostication, imaging analysis, and patient stratification, yet their clinical translation remains constrained by methodological shortcomings. Parsimonious models leveraging readily available clinical variables frequently rival more complex approaches, underscoring the importance of task-specific model selection. Future research should prioritize multicenter prospective datasets, external validation, calibration assessment, and evaluation of clinical impact to enable reliable integration of AI into TBI care.

## Introduction and background

Traumatic brain injury (TBI) is a major global health concern and a leading cause of death and long-term disability across all age groups. Millions of individuals experience TBI each year, resulting in substantial personal, social, and economic burdens [1]. Outcomes vary widely-from full recovery to severe disability or death-depending on injury severity, mechanism, and access to timely care, making prognostication and treatment decisions highly challenging [2].

Traditional assessment tools such as the Glasgow Coma Scale, neuroimaging classifications, and regression-based prediction models are widely used but limited in accuracy, as they fail to capture the nonlinear and dynamic processes underlying TBI [3]. These methods often rely on static admission data that do not reflect the evolving physiological state of the patient, contributing to persistent uncertainty in individualized prognostic assessment [4].

Artificial intelligence (AI) and machine learning (ML) have emerged as promising approaches to address these limitations. By analyzing large, multimodal datasets-including clinical, physiological, laboratory, and neuroimaging data-AI models can uncover complex relationships that may not be apparent through conventional analysis [5]. Applications in TBI include automated CT lesion detection, prediction of mortality and long-term functional outcomes, and estimation of treatment intensity [6].

Recent studies demonstrate that AI models can achieve strong predictive performance across clinical settings. Some leverage longitudinal ICU monitoring data for dynamic outcome prediction, while others show that simple triage-level clinical features can yield accurate mortality estimates. Imaging-based deep learning models, particularly those for CT segmentation, also show promise in supporting early treatment planning [7]. However, most studies are constrained by small sample sizes, retrospective single-center designs, inadequate handling of missing data, and lack of external validation, which limit generalizability [8].

Given the growing interest in this field, a systematic review is warranted to synthesize current evidence and evaluate the role of AI in TBI care. This review aims to summarize study populations, data sources, AI model characteristics, and outcomes, while critically assessing methodological quality and risk of bias. The findings will clarify current strengths and limitations, highlight research gaps, and guide future efforts toward clinically robust, interpretable, and generalizable AI applications in TBI.

## Review

Methodology

Literature Search Strategy

This systematic review followed the Preferred Reporting Items for Systematic Reviews and Meta-Analyses (PRISMA) guidelines [9]. A comprehensive search was conducted in PubMed, Web of Science, Scopus, and the Cochrane Central Register of Controlled Trials (CENTRAL) from inception to September 2025. Controlled vocabulary and free-text terms were combined, including (“traumatic brain injury” OR TBI OR “head injury” OR “brain trauma” OR “head trauma” OR “intracranial hemorrhage” OR “subdural” OR “epidural” OR “contusion”) AND (“artificial intelligence” OR AI OR “machine learning” OR “deep learning” OR “neural network*” OR “random forest” OR “support vector*” OR “transformer*” OR “foundation model*” OR “large language model*” OR LLM). Search strategies were tailored for each database, limited to English-language studies involving human participants, and supplemented by manual screening of reference lists from eligible publications.

Eligibility Criteria

Eligibility was defined using the PICO framework (Population, Intervention, Comparison, Outcomes, Study design) [10]. Studies were included if they: (1) involved patients with TBI or related intracranial injuries; (2) developed, validated, or applied AI or machine learning models for diagnosis, prognosis, monitoring, or treatment support; (3) reported a comparator such as a statistical model, clinical scale, or alternative AI algorithm; and (4) presented quantitative performance metrics (e.g., accuracy, sensitivity, specificity, AUC [area under curve]). Conference abstracts without full text, non-human studies, reviews, editorials, and studies not primarily focused on AI in TBI were excluded.

Study Selection and Data Extraction

Two reviewers independently screened titles and abstracts according to predefined criteria, retrieved full texts of potentially eligible articles, and resolved disagreements through discussion or, when needed, consultation with a third reviewer. Data extraction was conducted independently by two reviewers using a standardized form, capturing study characteristics, population and setting, data sources and modalities, AI model details, comparators, and reported performance outcomes. Discrepancies were resolved by consensus.

Quality Appraisal

Methodological quality and risk of bias were assessed using the Prediction model Risk of Bias Assessment Tool (PROBAST) [11], which evaluates four domains: participants, predictors, outcome, and analysis. Each domain was rated as low, high, or unclear risk of bias, with overall risk and applicability judgments determined by consensus.

Results

Study Selection

**Figure 1:**
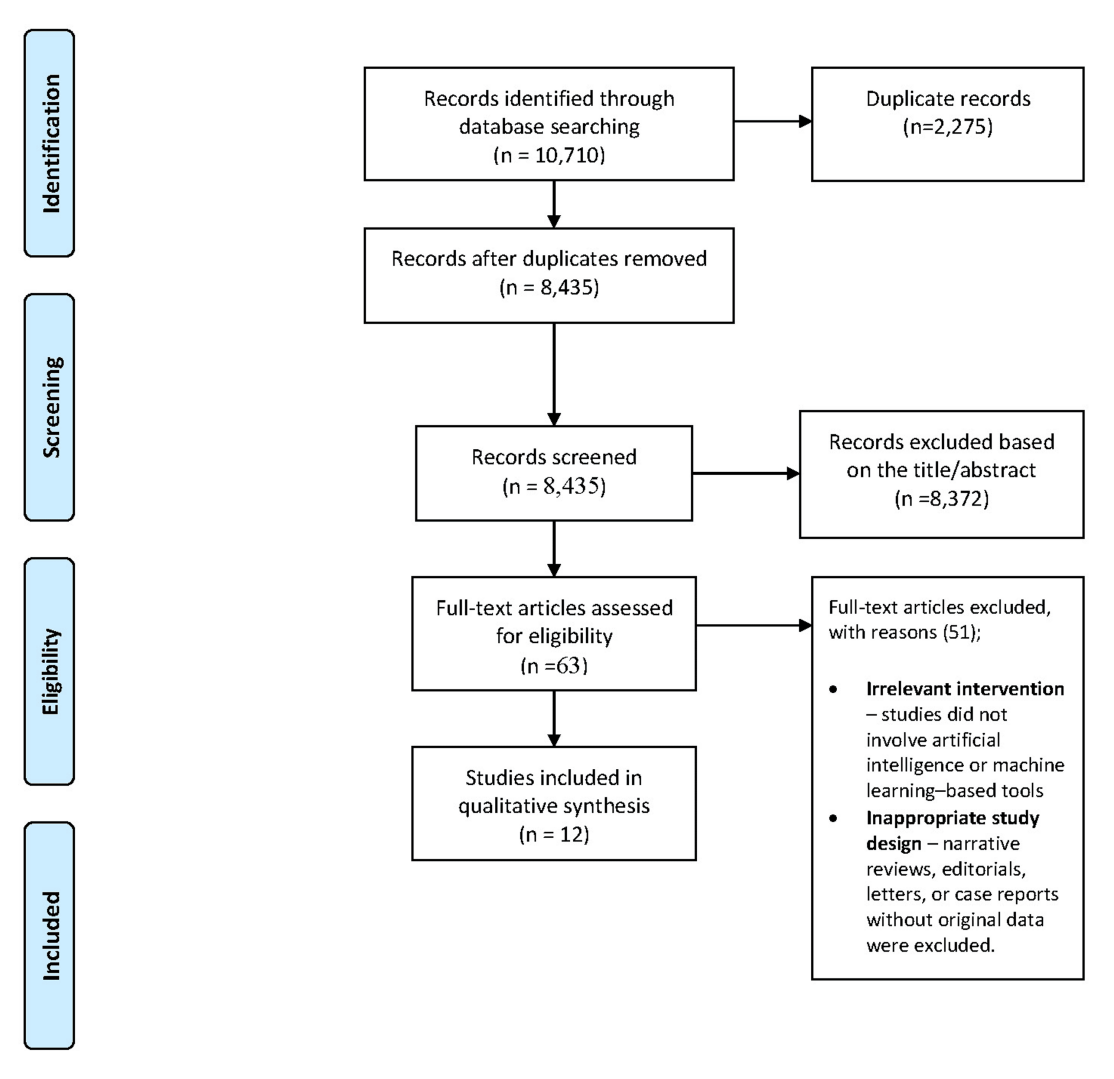
Flow diagram of the study selection process

A total of 10,710 records were identified across PubMed (n=3,913), Cochrane (n=565), Scopus (n=2,476), and Web of Science (n=3,756). No additional studies were found through manual searches or grey literature. After removing duplicates, 8,435 unique records remained for screening. Titles and abstracts were reviewed, and 8,372 records were excluded for irrelevance or failure to meet inclusion criteria. The full texts of 63 articles were assessed, and 51 were excluded due to unsuitable study design, lack of relevance, or insufficient data. Ultimately, 12 studies [12-23] met the eligibility criteria and were included in the qualitative synthesis (Figure 1).

Study Characteristics

The 12 included studies were conducted across North America, Europe, Asia, and the Middle East, encompassing designs such as multicenter prospective cohorts, registry-based analyses, and single-center retrospective studies (Table 1). Most focused on prognostic modeling using routine admission or triage variables (e.g., age, GCS, pupil reactivity, vitals, basic labs) in emergency or ICU settings. Examples include single-center models from Iran and China [14,20], a three-hospital triage cohort from Taiwan [23], a U.S. trauma registry analysis [13], and an ICU study from Qatar emphasizing coagulation and metabolic markers [17]. A Finnish multicenter study incorporated dynamic time-series ICU data to update risk every eight hours over five days [21]. Imaging-based investigations included a French prospective cohort using CNN-based CT segmentation to predict therapeutic intensity [12], a U.S. interpretable fuzzy-neural model combining radiology and baseline examination [18], and a pediatric MRI/DTI pilot study [15]. Two additional studies explored structure discovery and screening: an unsupervised phenotyping analysis with external replication [16] and an emergency department (ED) mild TBI screening model from Brazil [22].

**Table 1:** Summary of key characteristics and performance outcomes of studies applying artificial intelligence and machine learning in traumatic brain injury Abbreviations: AUC, area under the receiver operating characteristic curve; AMSE, average mean squared error; AD, axial diffusivity; AIS, Abbreviated Injury Scale; aPTT, activated partial thromboplastin time; BMI, body mass index; BS, blood sugar; BT, body temperature; CHOP, Children’s Hospital of Philadelphia; CNN, convolutional neural network; CPP, cerebral perfusion pressure; CV, cross-validation; DFA, discriminant function analysis; DTI, diffusion tensor imaging; ED, emergency department; EHR, electronic health record; FA, fractional anisotropy; FNR, false negative rate; FPR, false positive rate; GCS, Glasgow Coma Scale; GBM, gradient boosting machine; GFT, genetic fuzzy tree; GLM, generalized linear model; GLRM, generalized low-rank model; GOSE, Glasgow Outcome Scale–Extended; GRU-D, gated recurrent unit with decay; Hb, hemoglobin; HR, heart rate; ICP, intracranial pressure; ICU, intensive care unit; INR, international normalized ratio; ISS, Injury Severity Score; KNN, k-nearest neighbors; LASSO, least absolute shrinkage and selection operator; LLM, large language model; LR, logistic regression; MAP, mean arterial pressure; MD, mean diffusivity; ML, machine learning; MLP, multilayer perceptron; MRMR, minimum redundancy maximum relevance; MRI, magnetic resonance imaging; Naïve Bayes GLM, Naïve Bayes generalized linear model; PRBC, packed red blood cells; RF, random forest; RBF-SVM, radial basis function support vector machine; RD, radial diffusivity; RNN, recurrent neural network; ROC, receiver operating characteristic; ROSE, random over-sampling examples; SBP, systolic blood pressure; SMOTE, synthetic minority oversampling technique; SpO₂, peripheral oxygen saturation; SVM, support vector machine; TBI, traumatic brain injury; TIL, therapeutic intensity level; TQIP, Trauma Quality Improvement Program; TRACK-TBI, Transforming Research and Clinical Knowledge in TBI; TTAS, Taiwan Triage and Acuity Scale; XGB/XGBoost, extreme gradient boosting.

Study ID	Country	Study Design	Population & Setting	Data Sources & Modalities	AI Model Characteristics	Comparator	Outcomes & Performance
Brossard et al., 2023 [12]	France (University Hospital of Grenoble)	Prospective cohort with external validation	Dataset 1: 29 severe TBI patients, 84 admission/follow-up CTs; Dataset 2: 12 patients for external validation	CT scans with manual and CNN-based lesion segmentation; clinical data (age, GCS, MAP, Hb, antiaggregant use)	CNN segmentation (BLAST-CT, CNN2–CNN4 with transfer learning, 4-/7-class); PhotonAI ML pipeline	Manual segmentation and BLAST-CT baseline	Manual 7-class: AUC 0.89±0.17 for high therapeutic intensity (TIL≥11); CNN4 Dice≈0.64, accuracy 83% (10/12 external); BLAST-CT poor (AUC≈0.60).
Cao et al., 2023 [13]	USA (TQIP registry, 2013–2021)	Registry-based retrospective cohort; development & external validation	545,388 isolated severe TBI patients (AIS≥3, blunt trauma)	TQIP registry: demographics, comorbidities, admission status, clinical data	XGBoost-powered Cox regression; penalized Cox (LASSO, ridge, elastic net) for selection; SHAP interpretability	Penalized Cox regression (elastic net)	XGBoost-Cox C-index 0.896; LOS ≤5 days AUC 0.917, ≤20 days AUC 0.813; top predictors: GCS, age, AIS 5, hypotension, SpO₂, temperature, PRBC, cirrhosis, cancer.
Cui et al., 2021 [14]	China (Tangdu Hospital, Fourth Military Medical University)	Retrospective cohort; prognostic model development	230 TBI patients undergoing decompressive craniectomy (2015–2019)	Demographics, GCS, labs (D-dimer, INR, fibrinogen, glucose), CT findings (contusion volume, hemorrhage, midline shift, cistern status), perioperative data	Random Forest; logistic regression; SMOTE for class balance; univariate feature selection	Logistic regression	RF accuracy 0.810, sensitivity 0.833, specificity 0.800, AUC 0.830; LR AUC 0.765–0.843; RF superior overall.
Fleck et al., 2021 [15]	USA (Cincinnati Children’s Hospital)	Pilot prospective cohort; secondary analysis	43 adolescents (11–16 yrs): 23 mild TBI, 20 orthopedic controls	MRI + DTI (48 MRI, 192 DTI features: FA, MD, AD, RD)	Genetic Fuzzy Trees (GFT) with evolutionary tuning; 225 training runs, 80/20 split	Naïve Bayes, DFA, RBF-SVM, RF, Extra Trees, KNN	GFT: 100% training, 62.3% validation accuracy; sensitivity 59.4%, specificity 65.1%; outperformed all but SVM; identified DTI predictors of 1-week recovery.
Folweiler et al., 2020 [16]	USA (CHOP & University of Pennsylvania)	Secondary RCT analysis (COBRIT) + external validation (TRACK-TBI Pilot)	1,213 adult TBI patients (COBRIT); 599 (TRACK-TBI) for validation	156 baseline clinical, lab, and CT variables	Unsupervised ML: GLRM → PAM clustering; K-NN for validation	GCS-based classification	Identified 3 phenotypes (A–C) with distinct CT/lab profiles; phenotypes B/C stable in validation; better prognostic stratification than GCS.
Mekkodathil et al., 2023 [17]	Qatar (Hamad Trauma Center)	Retrospective cohort (2016–2021); model development & validation	922 hospitalized TBI patients (mean age 32; 94% male; 59% road traffic injuries)	Trauma registry + EHR; 13 admission features (GCS, ISS, coagulation, electrolytes, lactic acid)	SVM, LR, RF, XGBoost; 5-fold CV with hyperparameter tuning	Cross-model comparison	SVM best (AUC 0.86); LR 0.84; RF/XGB 0.85–0.86 but overfit; key predictors: aPTT, INR, PT, lactic acid, ISS, GCS, magnesium.
Minoccheri et al., 2022 [18]	USA (University of Michigan, multi-department)	Retrospective analysis; model development and validation	833 hospitalized TBI patients from the ProTECT III trial (excluding non-survivable injuries)	Admission EHR data (58 clinical and radiologic features; reduced to 18 via SHAP and MRMR)	Tropical geometry-based Fuzzy Neural Network (TFNN); interpretable rule extraction	XGBoost, Random Forest, SVM	TFNN AUC ≈ 0.799 (comparable to RF 0.810); accuracy ≈ 0.719; interpretable rules provided; robust across feature subsets.
Nayebi et al., 2021 [19]	USA (TRACK-TBI, 18 trauma centers)	Multicenter prospective study; model development and internal validation	902 ICU-admitted TBI patients (from 2996 TRACK-TBI participants); 6-month GOSE follow-up	110 features (59 static clinical, 51 time-series: vitals/labs over 5 ICU days)	Attention-based RNN with GRU-D units; interpretable via SHAP	IMPACT model (logistic regression)	RNN AUC 0.86 (95% CI 0.83–0.89) vs. IMPACT 0.69; superior across AUC, F1, AMSE, Kendall τ; time-series > static features.
Nourelahi et al., 2022 [20]	Iran (Rajaee Trauma Hospital, Shiraz University)	Retrospective cohort; model development and validation	2,381 severe TBI patients (1,682 after exclusions)	Admission data: age, sex, Rotterdam CT index, BS, SBP, GCS motor, PT-INR, pupils	Logistic Regression, Random Forest, SVM; feature selection via SFS and RF importance	LR, RF, SVM compared	All AUC ≈ 0.81–0.83; LR slightly best (AUC 0.83±0.05, sensitivity 0.78, specificity 0.78, accuracy 0.78); key predictors: GCS motor, pupils, age.
Raj et al., 2019 [21]	Finland (Helsinki, Kuopio, Turku University Hospitals)	Multicenter retrospective observational	472 adults admitted to ICU with ICP monitoring ≥24h (2003–2017)	High-frequency ICU data: ICP, MAP, CPP, GCS (motor, eye), baseline clinical data	ML-based logistic regression; dynamic models (ICP–MAP–CPP and ICP–MAP–CPP–GCS); updated every 8h for 5 days	IMPACT-TBI prognostic model	AUC increased from 0.67→0.81 (ICP–MAP–CPP) and 0.72→0.84 (ICP–MAP–CPP–GCS) from day 1→5; IMPACT-TBI AUC 0.78; good discrimination and calibration.
Terabe et al., 2023 [22]	Brazil (Regional University Hospital of Maringá)	Retrospective cross-sectional study	1,851 mild TBI patients (≥14 yrs) presenting to ED (2018)	EMR data; 10 clinical variables (mechanism, GCS, amnesia, dizziness, headache, vomiting, convulsion)	Six algorithms: RF, KNN, GBM, linear XGBoost, C5.0, Naïve Bayes GLM; ROSE balancing	Cross-model comparison	Accuracy 80–86%, specificity 80–87%, sensitivity 43–70%; linear XGBoost best (sensitivity 70%±0.06, AUC≈0.79, accuracy≈0.80); developed “Predict CT-Calculator.”
Tu et al., 2022 [23]	Taiwan (Chi Mei Medical Group, 3 hospitals)	Retrospective cohort; internal & external validation	18,249 adult TBI patients at ER triage (2010–2019); 200 external cases (2020)	12 triage variables: age, sex, BMI, TTAS, HR, BT, RR, GCS, pupil size/light reflex (L/R)	Six algorithms (LR, RF, SVM, LightGBM, XGBoost, MLP); SMOTE + 5-fold CV	Algorithm comparison	LR best: AUC 0.925 (95% CI 0.901–0.950), accuracy 0.893, sensitivity 0.812, specificity 0.894; external: sens. 100%, spec. 84.3%, acc. 84.5%; implemented as live web triage tool.

Methodologically, models spanned logistic regression, tree-based algorithms, support vector machines (SVM), and deep or hybrid architectures. Logistic regression often matched or exceeded complex models, particularly in large, heterogeneous datasets [13,20,23]. Tree-based methods (random forest, gradient boosting, LightGBM) were frequently tested against LR and SVM, though smaller or imbalanced samples sometimes produced overfitting [11,14]. Dynamic approaches improved discrimination with accumulating longitudinal data [21], while deep or hybrid designs-such as attention-based GRU-D networks [19], fuzzy-neural models [18], and CNN-derived CT segmentations [12]-were applied selectively. Unsupervised clustering identified phenotypes with distinct outcomes beyond GCS categories [15], and an adolescent MRI/DTI pilot explored Genetic Fuzzy Trees versus standard comparators [15]. A mild TBI screening prototype in the ED favored a linear XGBoost variant balancing accuracy and sensitivity [22].

Quality Assessment

Risk of bias varied substantially across studies due to differences in design, population representativeness, and analytical rigor. Several investigations [12,14,15,18] were rated as high risk because of small, single-center cohorts, retrospective design, or limited validation (Table 2). Moderate concerns were noted in studies such as [16,17,19-22], where predictors and outcomes were generally well defined, but internal validation, missing data handling, and single-center scope limited generalizability. By contrast, two large-scale investigations [13,22] demonstrated low risk of bias, supported by robust multicenter datasets, clearly defined predictors, rigorous external validation, and transparent reporting. Overall, while predictors and outcomes were consistently sound, participant selection and analysis domains often contributed to higher bias, emphasizing the need for larger, multicenter datasets and stronger validation frameworks.

**Table 2:** Summary of risk of bias assessment for included studies on AI-based models in traumatic brain injury prognostication Abbreviations: AI, artificial intelligence; ML, machine learning; TBI, traumatic brain injury; RoB, risk of bias; ICU, intensive care unit; CT, computed tomography; MRI, magnetic resonance imaging; CNN, convolutional neural network; DTI, diffusion tensor imaging; ICP, intracranial pressure; MAP, mean arterial pressure; CPP, cerebral perfusion pressure; GCS, Glasgow Coma Scale; GOSE, Glasgow Outcome Scale–Extended; GOS-E, Glasgow Outcome Scale–Extended; GRU-D, gated recurrent unit with decay; SHAP, Shapley additive explanations; MRMR, minimum redundancy maximum relevance; SMOTE, synthetic minority oversampling technique; CV, cross-validation; RCT, randomized controlled trial; EHR, electronic health record; SBP, systolic blood pressure; BS, blood sugar; PT-INR, prothrombin time–international normalized ratio; ISS, injury severity score; RNN, recurrent neural network; XGB, extreme gradient boosting; LR, logistic regression; RF, random forest; SVM, support vector machine; HIS, hospital information system; TTAS, Taiwan Triage and Acuity Scale; PCSS, Post-Concussion Symptom Scale; PSI, population stability index.

Study ID	Participants (RoB / Applicability)	Predictors (RoB / Applicability)	Outcome (RoB / Applicability)	Analysis (RoB)	Overall RoB
Brossard et al., 2023 [12]	High / Some concerns – Very small cohorts (29 and 12 patients); prospective but limited representativeness; high selection bias risk.	Low / Some concerns – Predictors were CT-derived lesion characteristics (volume, type, location) plus clinical data; segmentation reliability varied (manual vs. CNN, Dice <0.7).	Low / Low – Outcome (therapeutic intensity Level ≥11 within 8 ICU days) was clinically relevant and objectively measured.	High – Nested CV with tiny datasets increases instability; limited external validation (n=12); potential overfitting; unadjusted confounders; class imbalance.	High
Cao et al., 2023 [13]	Low / Low – Extremely large sample (n=545,388) from TQIP; highly representative of severe isolated TBI population.	Low / Low – Predictors (demographics, comorbidities, admission data) were well defined and routinely collected.	Low / Low – Outcome (in-hospital mortality) was objective and standardized.	Low – Proper training/test split (80/20); penalized regression and XGBoost with CV; imputed missing data; internal–external validation within dataset; transparent reporting (TRIPOD adhered).	Low
Cui et al., 2021 [14]	Some concerns / Some concerns – Single-center, retrospective; only TBI patients undergoing decompressive craniectomy included, limiting generalizability to broader TBI populations.	Low / Low – Predictors (demographics, labs, CT, perioperative factors) were standard, clinically relevant, and assessed before outcome.	Low / Low – Outcome (1-year mortality) was objectively defined and measured consistently via follow-up.	High – Small sample (n=230), overfitting risk (random forest nearly perfect on training but lower on test); SMOTE balancing may bias performance; only internal testing, no external validation.	High
Fleck et al., 2021 [15]	High / Some concerns – Very small pilot (n=43 adolescents); strict exclusions reduce representativeness.	Low / Some concerns – 240 imaging predictors (48 volumetric + 192 DTI) collected within 96h post-injury; measurement reliability not discussed.	Low / Low – Outcome (PCSS-defined 1-week recovery) was validated and prospectively measured.	High – Extremely high predictor-to-sample ratio; no external validation; unstable results (validation accuracy 62%).	High
Folweiler et al., 2020 [16]	Some concerns / Some concerns – Data from COBRIT (n=1,213) and TRACK-TBI Pilot (n=599); limited to CT-positive TBI, reducing generalizability.	Low / Low – 156 baseline features (demographics, labs, CT, physiology, tox) were well defined and pre-assessed before outcome.	Low / Low – Outcomes (GOS-E at 90 & 180 days) were validated and objective; missing outcomes were imputed or assigned for deaths.	Some concerns – Unsupervised clustering (GLRM + PAM); external validation with KNN not fully reproducible; feature selection data-driven; possible overfitting, though stability checks partly mitigate.	Some concerns
Mekkodathil et al., 2023 [17]	Low / Some concerns – National trauma registry (n=922, Qatar); representative but single-country; exclusions applied (penetrating injuries, transfers).	Low / Low – Thirteen bio-clinical predictors (labs, electrolytes, GCS, ISS) objectively measured at admission.	Low / Low – Outcome (in-hospital mortality) was objective and standardized.	Some concerns – Models (SVM, LR, RF, XGBoost) used internal 80/20 split and 5-fold CV; no external validation; RF and XGBoost showed overfitting; SVM robust.	Some concerns
Minoccheri et al., 2022 [18]	Some concerns / Some concerns – ProTECT III trial population skewed toward severe TBI; excluded non-survivable injuries, limiting generalizability.	Low / Low – Predictors (EHR and CT at admission) were clearly defined, assessed consistently, and measured before outcomes.	Low / Low – Outcome (6-month GOSE) was validated, clinically relevant, and assessed independently of predictors.	High – Only internal 10-fold CV, no external validation; data-driven feature selection (SHAP, MRMR) risks optimism; unclear handling of missing data.	High
Nayebi et al., 2021 [19]	Low / Low – Multicenter TRACK-TBI cohort (n=902) representative of a wide TBI spectrum; inclusion/exclusion criteria clear.	Low / Low – 110 predictors (59 static, 51 time-series) were well defined, collected prospectively, measured prior to outcomes.	Low / Low – Six-month GOSE was validated, clinically relevant; dichotomization justified.	Some concerns – 10-fold CV only; no external validation; potential optimism from complex RNN model; missingness handled by GRU-D, but residual bias possible.	Some concerns
Nourelahi et al., 2022 [20]	Some concerns / Some concerns – Large single-center (n=2,381) but retrospective; excluded ~29% with missing data, possibly biasing results.	Low / Low – Nine admission predictors (age, GCS motor, pupils, SBP, BS, PT-INR, Rotterdam CT index) were objective, standard, and pre-assessed prior to outcomes.	Low / Low – Six-month GOSE was well validated and assessed by experts.	Some concerns – Only internal 10-fold CV; no external validation; feature selection on same dataset (overfitting risk); missing data excluded rather than imputed.	Some concerns
Raj et al., 2019 [21]	Low / Some concerns – Multicenter ICU sample (n=472, Finland) enhances representativeness, but excluded patients with ICP monitoring <24h or death <36h, limiting generalizability.	Low / Low – ICP, MAP, CPP, and GCS (motor and eye) are standard ICU variables, measured consistently and blinded to outcomes.	Low / Low – Outcome (30-day mortality) was objective, time-bound, and obtained from a national registry.	Some concerns – Only internal CV; feature elimination recursive on full dataset (possible optimism); missing data not imputed; treatment interventions (e.g., decompression) not modeled, may bias predictions.	Some concerns
Terabe et al., 2023 [22]	Some concerns / Some concerns – Single-center (Brazil); retrospective; limited to 2018 cohort; uncertain generalizability.	Low / Low – Ten routine clinical predictors (mechanism, GCS, amnesia, headache, vomiting, etc.) were clearly defined and pre-assessed prior to outcomes.	Low / Low – Outcomes (positive head CT with neurosurgical relevance) were objective and clinically meaningful.	Some concerns – Six ML models compared with repeated CV and ROSE balancing; no external validation; class imbalance (~4.6% CT-positive) may bias sensitivity; best model (XGB) modest (70%).	Some concerns
Tu et al., 2022 [23]	Low / Low – Very large multicenter cohort (n=18,249) representative of ER triage population.	Low / Low – Twelve routine triage predictors (age, sex, BMI, TTAS, vitals, GCS, pupils) were clearly defined and pre-assessed prior to outcome.	Low / Low – Outcome (in-hospital mortality) was objective and validated.	Low – Six ML algorithms with proper train/test split (70/30), calibration, hyperparameter tuning; external validation (n=200) conducted; integrated into HIS.	Low

Prognostic Models for Mortality and Functional Outcomes

Across triage, ICU, and registry settings, most prognostic models achieved good to excellent discrimination using routine variables. A large multicenter triage study (n=18,249) showed that a logistic regression model with 12 features achieved AUC≈0.93 and was externally validated [22]. ICU-based models using admission labs reached AUCs of 0.84-0.86, though tree-based methods tended to overfit compared with LR or SVM. Coagulation and metabolic markers (aPTT, INR, PT, lactate, GCS) were consistently strong predictors [17]. Dynamic ICU models improved over time, with AUCs rising from ~0.70 on day 1 to ~0.83 by day 5 as longitudinal data accumulated [21]. Registry-scale analyses (n≈545k) combining penalized Cox and XGBoost achieved a C-index ≈of 0.90 overall [10]. Smaller single-center studies showed AUCs of 0.81-0.83, with LR often performing best [20].

Imaging-Based Prediction and Segmentation

CT-derived lesion volumes and locations-manually or via CNN segmentation, enhanced prediction of therapeutic intensity levels compared with clinical data alone. Transfer-learned models achieved an internal AUC≈of 0.89, with modest but supportive external validation [12]. Interpretable fuzzy-neural models integrating CT and examination data produced human-readable rules with discrimination comparable to RF and XGB [18]. While imaging features add predictive value, small sample sizes and variable segmentation quality (Dice <0.7) limit certainty.

Unsupervised Phenotyping and Subgroup Discovery

Data-driven clustering revealed clinically meaningful phenotypes not captured by GCS alone. Using six baseline labs (platelets, hematocrit, hemoglobin, PT, INR, glucose), three phenotypes showed distinct CT patterns and diverging GOS-E trajectories; two generalized to an external cohort [16].

Pediatric and Adolescent Cohorts

In adolescents with mild TBI, a high-dimensional MRI/DTI model achieved ~62% accuracy for 1-week recovery using Genetic Fuzzy Trees, performing comparably to SVM but limited by a small sample (n=43) and risk of overfitting [15]. Larger, externally validated pediatric cohorts are needed.

Screening Tools for Mild TBI

In an ED cohort (n=1,851), classifiers reached an accuracy of ~0.80-0.86 but modest sensitivity (~0.70) for CT-positive findings. A linear XGBoost variant achieved the best sensitivity and was implemented as a “Predict CT-Calculator” prototype [22]. Given the low prevalence (~5%) of CT-positive cases, calibration and cost-sensitive evaluation are critical before clinical use.

Cross-Cutting Themes: Validation, Interpretability, and Generalizability

External validation was limited in most studies, with performance often declining in independent datasets. Compact, clinically intuitive models using common features (age, GCS, pupils, vitals, simple labs) frequently matched or outperformed complex architectures [20,23]. Transparent modeling approaches, such as LR with SHAP values and fuzzy-neural rules, enhanced interpretability without major loss of accuracy [13,15]. However, small, retrospective designs and exclusion due to missing data introduced potential bias [20]. Finally, model performance varied by time horizon; short-term predictions performed best in registry studies [13], while dynamic ICU models improved with accumulating data [21].

## Conclusions

This systematic review underscores the expanding role of artificial intelligence in traumatic brain injury management. Current evidence shows that AI-based models can predict mortality, functional outcomes, imaging findings, and screening results with high accuracy, often comparable to or surpassing traditional methods. Notably, parsimonious models using routine clinical features frequently perform as well as more complex algorithms, highlighting that model sophistication should align with data quality and context. Nonetheless, broad clinical adoption remains limited by small single-center samples, methodological bias, inadequate external validation, and poor reporting of calibration and decision-curve analyses. Advancing the field will require large multicenter prospective studies, robust validation, transparent reporting, and interpretable models. Addressing these gaps could enable AI to become a reliable tool for prognostication, individualized care, and clinical decision support in TBI.
